# Complexity-Based Analysis of the Variations of Brain and Muscle Reactions in Walking and Standing Balance While Receiving Different Perturbations

**DOI:** 10.3389/fnhum.2021.749082

**Published:** 2021-10-08

**Authors:** Najmeh Pakniyat, Hamidreza Namazi

**Affiliations:** ^1^Independent Researcher, Toronto, ON, Canada; ^2^Incubator of Kinanthropology Research, Faculty of Sports Studies, Masaryk University, Brno, Czechia; ^3^College of Engineering and Science, Victoria University, Melbourne, VIC, Australia

**Keywords:** muscle, brain, EEG signals, EMG signals, complexity, walking, standing, perturbations

## Abstract

In this article, we evaluated the variations of the brain and muscle activations while subjects are exposed to different perturbations to walking and standing balance. Since EEG and EMG signals have complex structures, we utilized the complexity-based analysis. Specifically, we analyzed the fractal dimension and sample entropy of Electroencephalogram (EEG) and Electromyogram (EMG) signals while subjects walked and stood, and received different perturbations in the form of pulling and rotation (via virtual reality). The results showed that the complexity of EEG signals was higher in walking than standing as the result of different perturbations. However, the complexity of EMG signals was higher in standing than walking as the result of different perturbations. Therefore, the alterations in the complexity of EEG and EMG signals are inversely correlated. This analysis could be extended to investigate simultaneous variations of rhythmic patterns of other physiological signals while subjects perform different activities.

## Introduction

Analysis of the alterations in human physiology during different locomotion is very important in sport sciences. Due to the changes in leg muscle activation, while doing different locomotions, many works analyzed EMG signals using various techniques (Oliveira et al., 2014; Subbu et al., 2015; Nazmi et al., 2019; Wang et al., 2019). Besides, since brain activation also changes in different locomotions, many studies worked on the analysis of EEG signals using various techniques (Presacco et al., 2011; Maidan et al., 2019; Bodda et al., 2020; Tortora et al., 2020).

Since the human brain controls muscle activations in different conditions, the variations in muscle and brain activations should be related. Therefore, we hypothesize that the characteristics of EMG and EEG signals should be correlated.

According to the literature, some studies have focused on simultaneous analysis of EEG and EMG signals during walking/running. These studies benefited from different techniques (e.g., frequency and amplitude analyses) to simultaneously analyze the alterations of EEG and EMG signals in case of normal subjects (Petersen et al., 2012; Artoni et al., 2017) and patients with movements disorders (e.g., Parkinson) (Günther et al., 2019; Roeder et al., 2020), during different types of locomotion (e.g., normal walking, stereotyped walking, and treadmill walking).

Besides all reported works in this area that analyzed brain and leg muscle reactions while performing different movements, no work has been reported that considered the complex structure of these signals for its analysis.

The activation of muscle and brain in the form of EMG and EEG signals have complex structures (Kumarasinghe et al., 2021; Soundirarajan et al., 2021). In fact, complexity is a concept to characterize the behavior of a system that contains many parts which interacting together in a highly variable way (Steven, 2001). Therefore, we can use the fractal theory to quantify their complex structures. Fractal objects have self-similar or self-affine structures that are distributed on every scale inside them. Self-similar fractals have the same scaling in different directions. However, self-affine fractals (e.g., EEG and EMG signals) (Namazi et al., 2021c) are not necessarily identical in different directions. The scaling rules are characterized by “scaling exponents” (dimension). The scaling exponent (ℵ) of fractals is related to their topological dimension (*D_T_*) based on Szpilrajn inequality:


(1) $$ℵ>D_{T}$$

Many studies have worked on the fractal analysis of biological and physiological time series [e.g., MEG signals (Namazi and Jafari, 2019), R-R time series (Sen and McGill, 2018), GSR signals (Namazi et al., 2021d), RNA random walks (Namazi et al., 2021e)]. However, limited studies have been conducted on the fractal analysis of leg muscle EMG signals while doing various movements. For instance, the reported studies on the fractal analysis on EMG signals which evaluated the coupling among the complexities of leg muscle activations and walking paths (Kamal et al., 2020), analyzed muscle reaction in patients with Parkinson’s disease (Ravier et al., 2016), investigated the reaction of vastus lateralis muscle during exercise routine (Garavito et al., 2016), and investigated the fatigue in cycling exercises (Beretta-Piccoli et al., 2018) can be mentioned.

Although the fractal theory has been widely applied in the analysis of the complexity of EEG signals in different conditions [e.g., external stimulation (Babini et al., 2020), detection of brain disorders (Namazi et al., 2020)], however, based on our search, only one reported work analyzed EEG signals during locomotion using fractal theory. In (Mujib Kamal et al., 2020), we showed that the variations of the complexity of EEG signals and walking paths are correlated. In other words, the complexity of EEG signals changes greater if a human walks on a path that is more complex.

The complexity of signals also can be quantified using other methods (e.g., sample entropy and approximate entropy). Sample entropy quantifies the complexity of time series, and it is independent of the data length (Namazi, 2020). Since the recorded signals from various participants were short (0.5–1 s in different conditions) and had various lengths, sample entropy is used in this study to validate the fractal analysis results. Several works quantified the complexity of EMG signals in various locomotions using sample entropy. For instance, the works that investigated the effect of walking speed (Namazi, 2021) and aging (Kang and Dingwell, 2016) on the complexity of leg muscle reaction, and classified Knee osteoarthritis (KOA) in walking at a self-paced speed (Chen et al., 2019), can be mentioned.

Although many reported works analyzed the complex structure of EEG signals in different conditions [e.g., response to different stimuli (Namazi et al., 2021a), classifying brain disorders (Simons et al., 2015)] using sample entropy, however, no reported work evaluated the complexity of EEG signals during walking or running using sample entropy.

Since no work has analyzed the complexity of EEG and EMG signals simultaneously during walking/running, we utilized fractal theory and sample entropy to evaluate the synchronization of the changes in EEG and EMG signals at different standing and walking conditions.

## Materials and Methods

We investigated the variations of brain and leg muscle reactions in walking and standing while subjects received different perturbations in the form of pulling and visual rotation. Since EEG and EMG signals are complex, we ran fractal analysis and computed their fractal dimension to quantify their complexity.

We used the box-counting algorithm for our analysis (Mat Dawi et al., 2021). It uses a series of same-size (μ) boxes to cover the time series. After that, the number of these boxes (*n*) is counted. In several steps, the size of these boxes is changed, and finally, and the fractal dimension (*FD*) is computed as:


(2) FD=limμ→0log⁡n(μ)log/1μ

*FD* in the general form is formulated in eq. 3, in which, *e* is the order of *FD*, and *p*_*i*_ indicates the probability (Mat Dawi et al., 2021).


(3) $$FD_{e}=\lim_{\text{x}\to 0}\frac{1}{e-1}\frac{\log\sum_{\text{i}=1}^{\text{n}}p_{i}^{e}}{\log\text{x}}$$

We also chose sample entropy to quantify the complexity of signals. It is known that the length of data does not affect the value of the sample entropy (Namazi et al., 2021b). Since the recorded EMG and EEG signals from different subjects were short (0.5–1 s) and had various lengths, sample entropy helped us verify the fractal analysis results.

For a time series in the form of {*y*(1), *y*(2), *y*(3), …, *y*(*n*)}, *Y*_*z*_(*i*) = {*y*_*i*_, *y*_*i* + 1_, *y*_*i* + 2_, …, *y*_*i* + *z*−1_} is defined as a template vector and the distance function *d*[*Y*_*z*_(*i*), *Y*_*z*_(*j*)](*i* ≠ *j*) is to be Chebyshev distance. Then, the sample entropy (*SamEn*) is formulated as (Delgado-Bonal and Marshak, 2019):


(4) $$SamEn=-log\frac{D}{E}$$

Considering ε as the tolerance (0.2 × *standard deviation of data*), *D* and *E* indicate the number of template vector pairs with the condition in (5) and (6):


(5) $$d\left[Y_{z+1}\left(i\right),Y_{z+1}\left(j\right)\right]<\varepsilon$$

(6) $$d\left[Y_{z}\left(i\right),Y_{z}\left(j\right)\right]<\varepsilon$$

We computed the fractal exponent and sample entropy of EEG and EMG signals to evaluate the simultaneous alterations of the brain and muscle reactions at different perturbations while subjects stood and walked.

### Database and Analysis

In this research, we used the open-access database provided by Peterson & Ferris which is available in Mobile Brain (2021). Their study has been approved by the University of Michigan Health Sciences and Behavioral Sciences Institutional Review Board. All subjects provided written informed consent before commencing the experiment.

**FIGURE 1:**
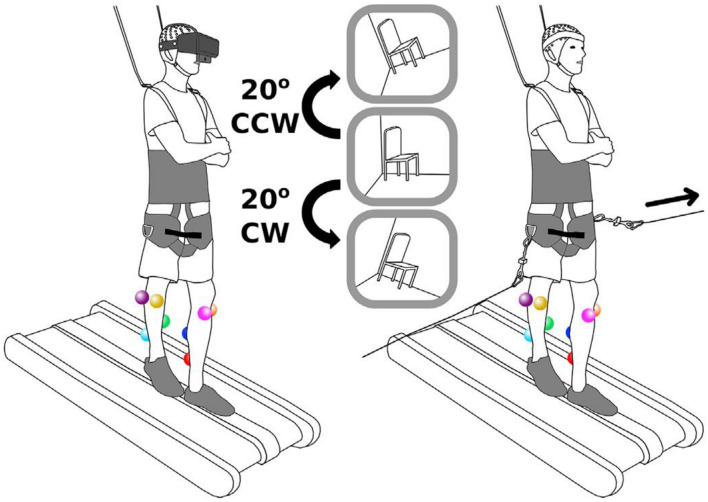
Schematics of the experiment (Peterson and Ferris, 2019).

This database includes the simultaneously recorded EEG, EMG, EOG, and sacrum/head position data for 30 healthy subjects (15 M, 15 F, 22.5 ± 4.8 years). The lower leg EMG signals (Vicon, Los Angeles, CA) were recorded at 1000 Hz from four electrodes attached to each leg of subjects including LTA (left tibialis anterior), LSOL (left soleus), LMG (left medial gastrocnemius), LPL (left peroneus longus), RTA (right tibialis anterior), RSOL (right soleus), RMG (right medial gastrocnemius), and RPL (right peroneus longus). The approximate locations of EMG electrodes are shown in Figure 1. The EEG signals (BioSemi Active II, BioSemi, Amsterdam, NL, United States) were recorded at 512 Hz from 128 channels.

In the experiment, participants walked at 0.22 m/s or stood on a 2.5 cm tall by 12.7 cm wide balance beam mounted to a treadmill. Figure 1 shows the schematics of the experiment. As can be seen in this figure, subjects wore a body-support harness for safety. Before starting the experiment, subjects were introduced to the experiment. Subjects were avoided from looking around and rotating across their body’s longitudinal axis.

Subjects were presented with two types of sensorimotor perturbations including a side-to-side pull at the waist, and a 20-degree field-of-view rotation (Figure 1). Two rotational motors on the sides of subjects were used for the side-to-side pull. As shown in Figure 1, subjects were pulled using a bar (connected to subjects through a wire) that was connected to one motor. This perturbation lasted for 1 s. At the end of perturbation, the motor rotated back to its starting position. Peterson & Ferris used virtual reality (VR) headset with an attached webcam to rotate the field of view of subjects. In fact, the perturbations included rotating the view of subjects 20° clockwise or counterclockwise through VR experience. The rotation of view lasted for 0.5 s before the view returned to its starting position.

Therefore, four conditions were applied on subjects as the combination of the two perturbation types and two physical tasks. It should be noted that each condition lasted 10 min, and subjects experienced 150 perturbations (75 per side, randomized). Please refer to Peterson and Ferris (2019) for more information about the experiment.

Peterson and Ferris (2019) synchronized the EMG and EEG data using a 0.5 Hz square wave. Initially, they downsampled EEG signals to 256 Hz, and then applied a high-pass filter (1 Hz) to the data. They also merged the EEG data across all conditions, referenced to the median channel value for each time point, and removed 60 Hz line noise. They also applied a 1 Hz high pass filter to EMG signals. For understanding the full steps for the pre-processing of signals, please refer to (Peterson and Ferris, 2019).

**FIGURE 2:**
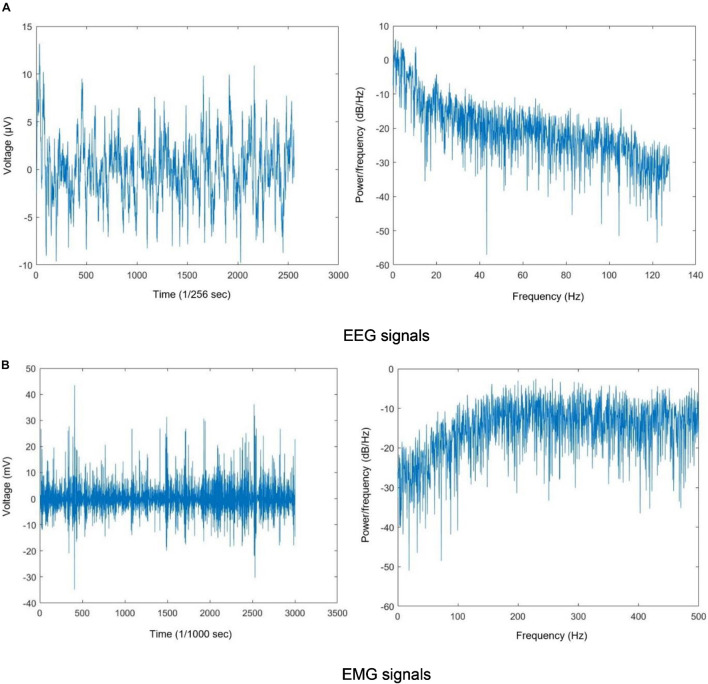
**(A)** EEG signals and **(B)** EMG signals. The filtered EEG and EMG signals and their periodogram PSD estimate.

Figures 2A,B show sample filtered EEG and EMG signals and their frequency information for a participant. These figures show 10 s of EEG data and 3 s of EMG data for better visibility.

We calculated the fractal dimension and sample entropy of filtered signals in the case of both legs in various conditions. The box-counting algorithm was run with the box sizes of $\frac{1}{2},\frac{1}{4},\frac{1}{8},\frac{1}{16},$. The smallest box size was chosen in the algorithm (Box Counting Algorithm, 2021). The length of the template vector was equal to the embedding dimension (=2). MATLAB R2020b was chosen for our analysis.

The Anderson-Darling test was chosen for checking the normality of results. The test’s result indicated the normal distribution of data. We compared the changes in the complexity of signals between standing and walking while pulling and rotating by running the student *t*-test (α = 0.05).

## Results

**FIGURE 3:**
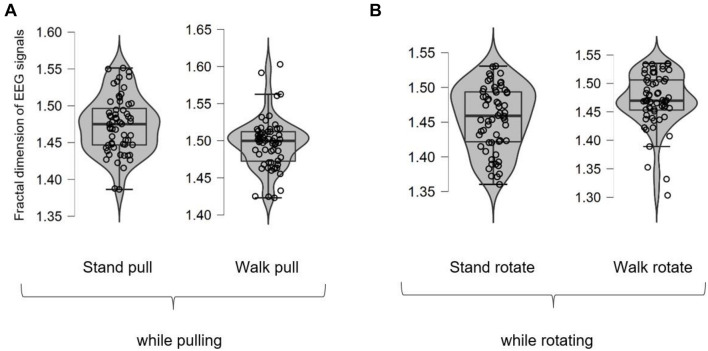
The box plots for the fractal dimension of EEG signals in walking and standing while pulling **(A)** and rotating **(B)**.

Figure 3 shows different box plots (with whiskers) for the fractal exponent of EEG signals in walking and standing while pulling and rotating. As shown in the case of each plot, the box plot includes a violin element that indicates the probability density of the data at different values. Besides, we also added the jitter elements to each plot to show the distribution of the data. As it is clear in these plots, only five outliers were identified (in the case of walk pull and walk rotate) which demonstrates the suitability of recorded data for inclusion in the analysis.

**FIGURE 4:**
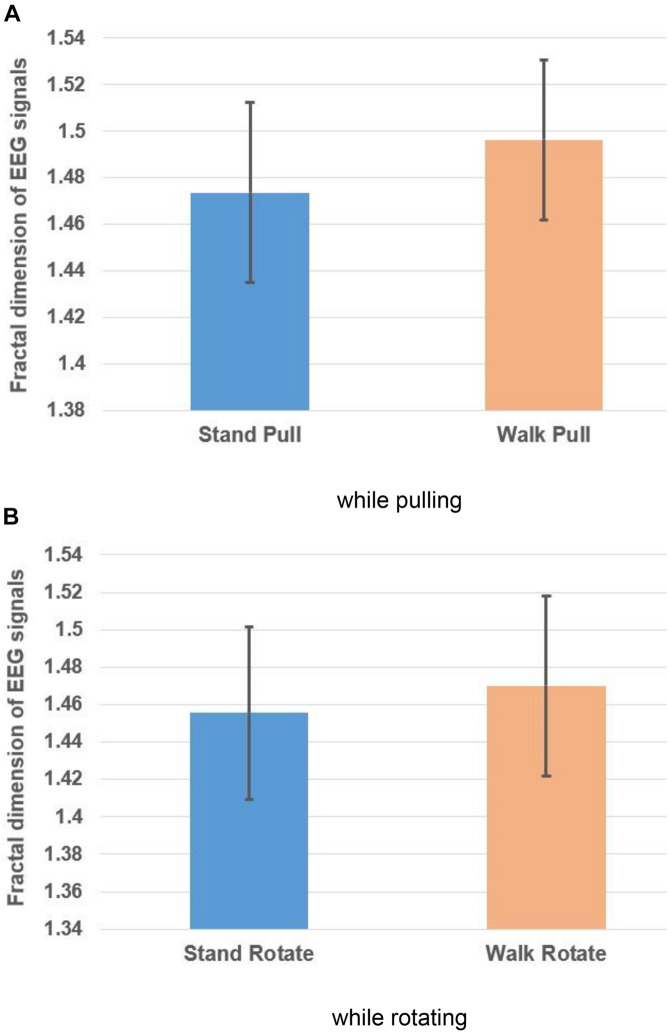
The fractal dimension of EEG signals in walking and standing while pulling **(A)** and rotating **(B)**. Error bars indicate standard deviation.

Besides, the averaged fractal exponent in walking and standing while pulling and rotating are shown in Figures 4A,B, respectively.

As Figures 4A,B illustrate, in the case of pulling and rotating, the fractal exponent of EEG signals has bigger values in walking than standing. Therefore, we can state that pulling and rotating of subjects while walking had bigger effects on the changes in the complexity compared to pulling and rotating while subjects stand. Since while walking there are more cognitive loads on subjects, the complexity of their EEG signals increased.

Table 1 lists the results of the student *t*-test which indicates the significant alterations in the fractal exponent among walking and standing conditions in case of pulling and rotating. In other words, walking caused a significant increase in the complexity of EEG signals in pulling and rotating. Besides, pulling of subjects caused a more significant alteration in the complexity of signals among walking and standing than rotating of them.

**TABLE 1:** Comparison of the fractal dimension of EEG signals between walking and standing in case of pulling and rotating.

Comparison	*p*-value
Stand pull vs. Walk pull	0.0004
Stand rotate vs. Walk rotate	0.0463

**FIGURE 5:**
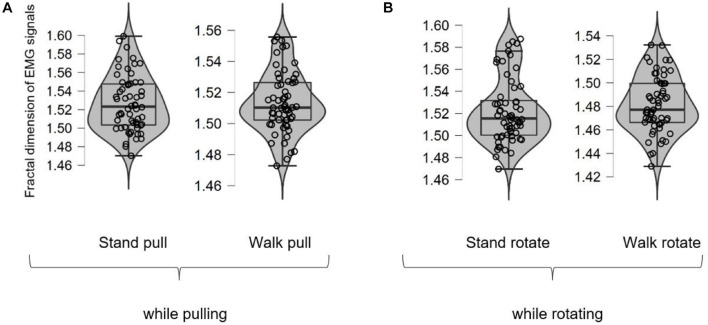
The box plots for the fractal exponent of EMG signals in walking and standing while pulling **(A)** and rotating **(B)**.

Figure 5 shows different box plots (with violin and jitter elements) for the fractal exponent of EMG signals in walking and standing while pulling (a) and rotating (b) in the case of different subjects. As it is clear in these plots, only four outliers were identified (in the case of stand rotate) which demonstrates the suitability of recorded data for inclusion in the analysis.

**FIGURE 6:**
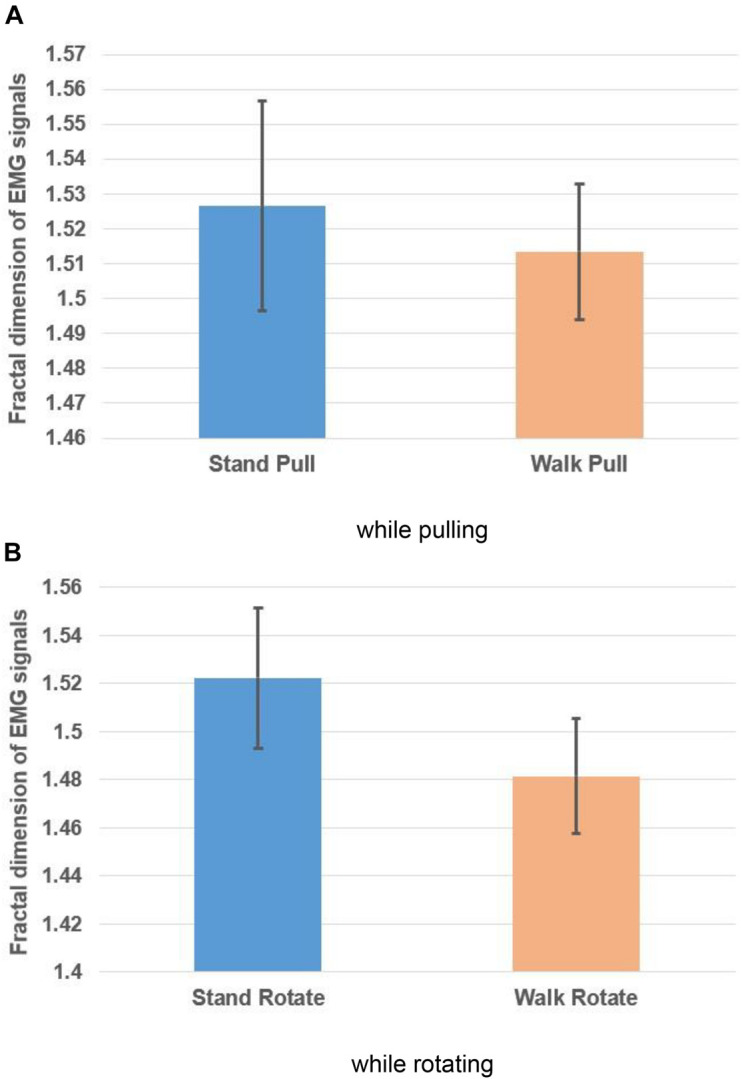
The fractal exponent of EMG signals in walking and standing while pulling **(A)** and rotating **(B)**. Error bars indicate standard deviation.

Figures 6A,B, respectively, illustrate the averaged alterations of the fractal exponent of EMG signals in walking and standing while pulling (a) and rotating (b).

As Figures 6A,B illustrate, in the case of pulling and rotating, the fractal exponent of EMG signals has bigger values in standing than walking. Therefore, we can state that EMG signals of subjects had lower complexity in walking than standing while pulling and rotating. Comparing these results with the presented results in Figures 4A,B indicate reverse trends. In other words, although the complexity of brain reactions increases in walking than standing, the complexity of muscle reaction decreases.

We also compared the alterations in the fractal exponent among walking and standing (in pulling and rotating) using the student *t*-test, and the results are brought in Table 2. As shown in Table 2, the results indicate significant alterations in the fractal exponent among walking and standing conditions in the case of pulling and rotating. In other words, walking caused a significant decrease in the complexity in pulling and rotating conditions. Comparing the results in Tables 2, 3 indicates that brain and leg muscles show significant differences in their reactions between walking and standing.

**TABLE 2:** Comparison of the fractal exponent of EMG signals between walking and standing in case of pulling and rotating.

Comparison	*p*-value
Stand pull vs. Walk pull	0.0025
Stand rotate vs. Walk rotate	0.0001

**TABLE 3:** Comparison of the sample entropy of EEG signals between walking and standing in case of pulling and rotating.

Comparison	*p*-value
Stand pull vs. Walk pull	0.0001
Stand rotate vs. Walk rotate	0.4382

**FIGURE 7:**
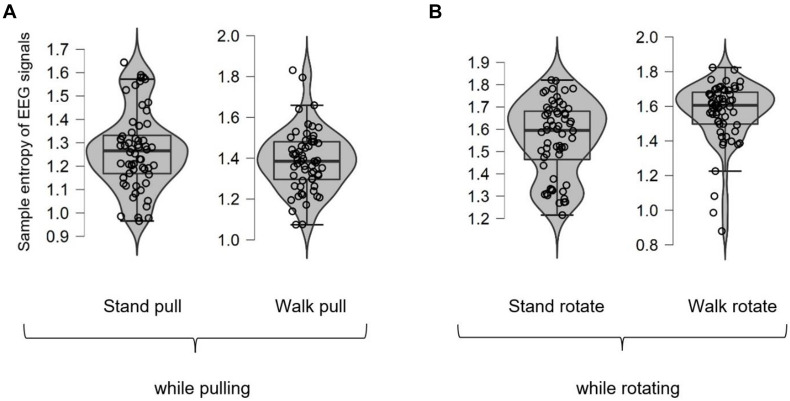
The box plots for the sample entropy of EEG signals in walking and standing while pulling **(A)** and rotating **(B)**.

As was mentioned previously, we also computed the sample entropy of EEG and EMG signals to verify the results of fractal analysis. Figure 7 shows different box plots (with violin and jitter elements) for the sample entropy of EEG signals in walking and standing while pulling (a) and rotating (b) in the case of different subjects. As it is clear in these plots, only six outliers were identified (in the case of stand pull, walk pull, and walk rotate) which demonstrates the suitability of recorded data for inclusion in the analysis.

**FIGURE 8:**
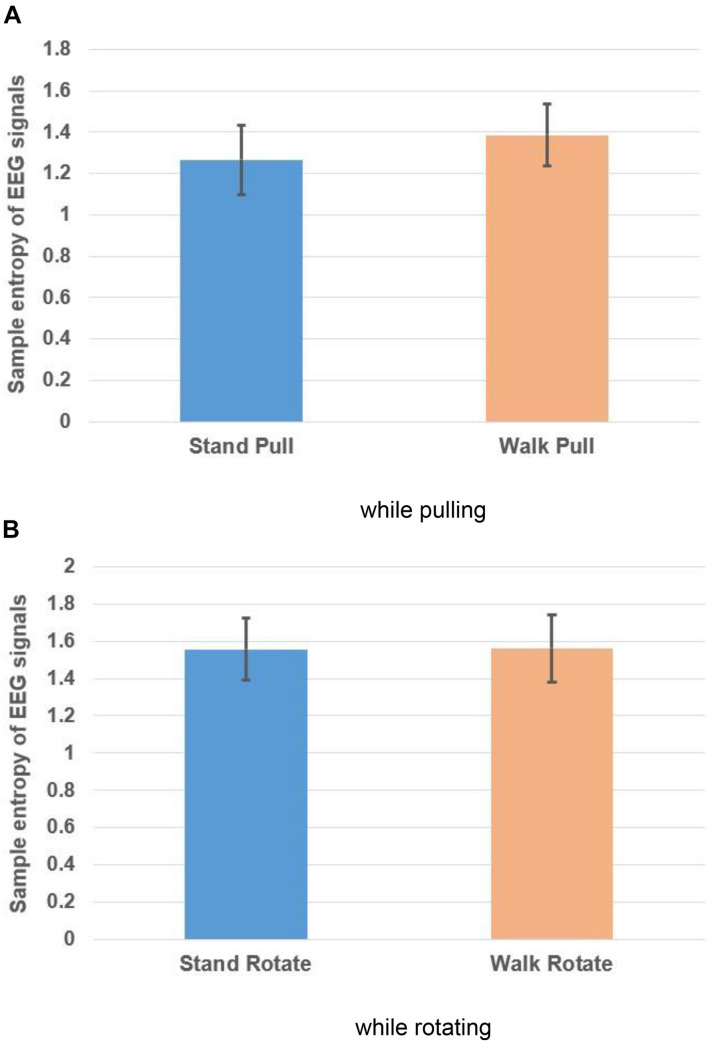
Sample entropy of EEG signals in walking and standing while pulling **(A)** and rotating **(B)**. Error bars indicate standard deviation.

Figures 8A,B, respectively, illustrate the variations of the sample entropy of EEG signals in walking and standing while pulling (a) and rotating (b).

As shown in Figures 8A,B, in the case of pulling and rotating, the entropy of EEG signals has bigger values in walking than standing. In other words, pulling, and rotating of subjects while walking caused bigger changes in the complexity compared to pulling and rotating while subjects stand. Comparing these results with the presented results in Figure 4 indicates that the result of analysis of entropy verified the fractal analysis results.

We also computed the *p*-values using the student *t*-test for comparing the variations in the sample entropy among walking and standing conditions in case of pulling and rotating. As brought in Table 3, there is a significant alteration in the sample entropy among walking and standing while pulling of subjects. However, the alterations in the entropy among standing and walking while rotating are not significant. Besides, similar to the results in Table 1, pulling of subjects caused a more significant change in the complexity among walking and standing than rotating of them.

**FIGURE 9:**
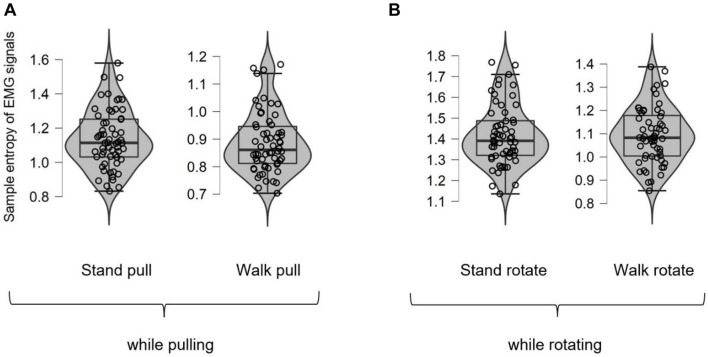
The box plots for the sample entropy of EMG signals in walking and standing while pulling **(A)** and rotating **(B)**.

Figure 9 shows different box plots (with violin and jitter elements) for the sample entropy of EMG signals in walking and standing while pulling (a) and rotating (b). As it is clear in these plots, only five outliers were identified (in the case of walk pull and stand rotate) which demonstrates the suitability of recorded data for inclusion in the analysis.

**FIGURE 10:**
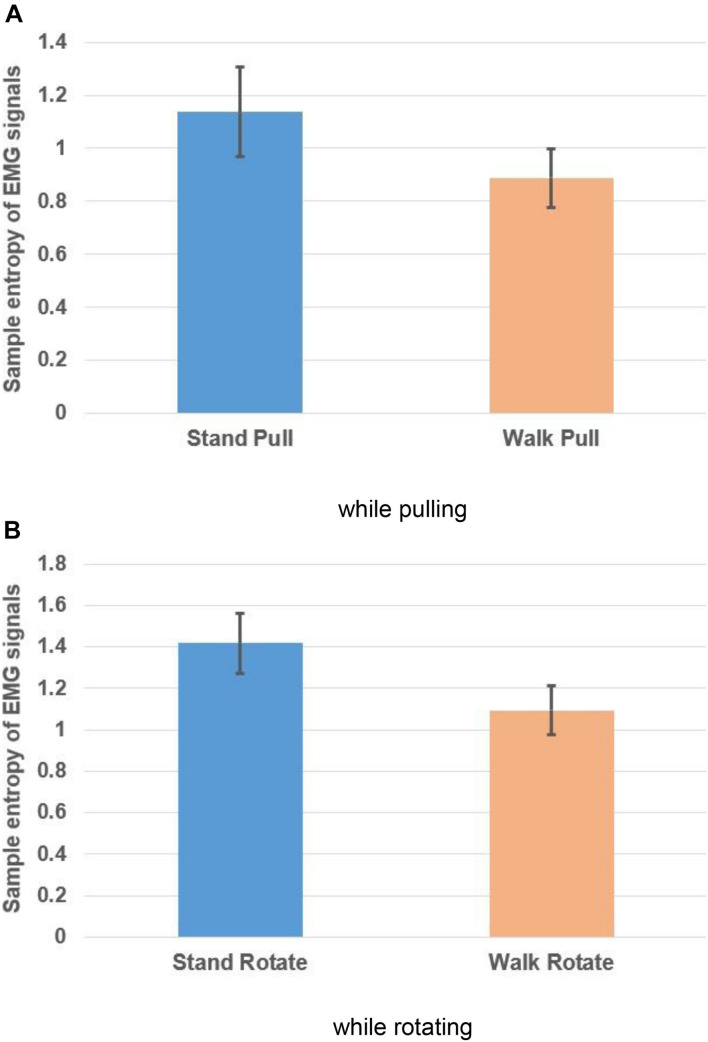
Sample entropy of EMG signals in walking and standing while pulling **(A)** and rotating **(B)**. Error bars indicate standard deviation.

Figures 10A,B, respectively, show the variations of the sample entropy of EMG signals in walking and standing while pulling (a) and rotating (b).

As Figures 10A,B illustrate, the entropy has bigger values in standing than walking in the case of pulling and rotating. Therefore, we can state that EMG signals of subjects had lower complexity in walking than standing while pulling and rotating. Comparing these results with the presented results in Figures 6A,B indicates that the result of entropy verified the fractal analysis results. Besides, comparing these results with the presented results in Figures 8A,B indicate reverse trends. In other words, although the complexity of brain reactions increases in walking than standing, the complexity of muscle reaction decreases.

Table 4 compares the entropy of EMG signals among walking and standing (in pulling and rotating) using the student *t*-test. The results demonstrated the significant alterations in the entropy among walking and standing conditions in case of pulling and rotating. In other words, similar to the presented results in Table 2, walking caused a significant decrease in the EMG signals’ complexity in both pulling and rotating conditions.

**TABLE 4:** Comparison of the sample entropy of EMG signals between walking and standing in case of pulling and rotating.

Comparison	*p*-value
Stand pull vs. Walk pull	0.0001
Stand rotate vs. Walk rotate	0.0001

Therefore, EEG signals have greater complexity in walking than rotating, however, EMG signals are less complex in walking than rotating.

## Conclusion and Discussion

We analyzed the changes in the brain and leg muscle reactions while subjects walked and stood and received different perturbations (in the form of pulling and rotation) by computing the fractal exponent and sample entropy of EEG and EMG signals.

The results demonstrated that the fractal exponent of EEG signals had greater values in walking than standing while subjects received perturbations. However, the result of the analysis of the fractal exponent of EMG signals showed a reverse trend compared to the obtained results for EEG signals. The analysis of the sample entropy of signals demonstrated similar results with the fractal analysis results. EEG signals have greater sample entropy in walking than standing whereas, the sample entropy of EMG signals has greater values in standing than walking. The result of statistical analysis also supported the obtained results.

Therefore, it can be concluded that the changes in brain and muscle reactions are inversely correlated. These results indicate the importance of complexity-based analysis for finding the connection among brain and muscle activations.

It is known that the brain controls muscle activation through the physiological network of the human body (Bashan et al., 2012). When we are standing or walking, the activity of leg muscles is controlled by the brain through the electrical impulses to the neuromuscular junction. Accordingly, electrical signals are converted into chemical signals allowing for muscle contraction. Therefore, leg muscle and brain activations should be related. The physiological aspect of the observed reverse correlation in this research can be investigated more by simultaneously considering the biological activation of leg muscles and the neural aspect of the brain’s control on leg muscles.

Here, we should note that the inverse relationship among the alterations of the complexity of EEG and EMG signals does not affect the importance of the results. Based on the literature, some researchers reported an increment in the EMG signals’ complexity in walking than standing (Kamal et al., 2020), whereas some other works found a decrement in the complexity of EMG signals as the walking speed increases (Namazi, 2021). Besides, the observed increment in the EEG signals’ complexity in walking is valid according to the provided results in Mujib Kamal et al. (2020). The main important point about these findings is that both fractal theory and sample entropy showed similar results. The findings of this study may challenge the conclusions drawn from other studies (Tao et al., 2015; Flood et al., 2019) that were based on choosing only one technique to quantify the complexity of EEG and EMG signals during walking or other activities.

The obtained results in this study have direct benefits in sport/physiological sciences, when we can link muscle activations to motor control and therefore, understand how the brain controls muscle’s activations in various movements in different sports.

We can extend our analyses to other conditions (e.g., walking at various speeds and inclines) to decode the correlation of the EEG-EMG signals in those conditions. Similarly, we can conduct our investigations for patients with various brain [e.g., Parkinson’s (Cantello et al., 1995)] and/or movement [e.g., motor stereotypies (Houdayer et al., 2014)] disorders to decode the EEG-EMG signals correlation.

The reported investigation in this study can be extended to evaluate the changes in human physiology while doing standing and walking (or any other movements) and receiving different perturbations. For this purpose, we can apply the complexity-based analysis on the related physiological signals. For instance, since walking affects our respiration rate, we can simultaneously analyze the variations of respiration time series and EEG signals at different perturbations. Since various organs are working together within the physiological network (Bashan et al., 2012) and are controlled by the brain, a correlation should exist among their related physiological signals. All these analyses are very important in physiological sciences.

## Data Availability Statement

Publicly available datasets were analyzed in this study. This data can be found here: https://figshare.com/articles/dataset/Mobile_brain_body_imaging_MoBI_during_sensorimotor_balance_perturbations/14175573.

## Ethics Statement

The studies involving human participants were reviewed and approved by University of Michigan Health Sciences and Behavioral Sciences Institutional Review Board. The patients/participants provided their written informed consent to participate in this study.

## Author Contributions

HN ran the analysis and drafted the manuscript. Both authors contributed to the article and approved the submitted version.

## Conflict of Interest

The authors declare that the research was conducted in the absence of any commercial or financial relationships that could be construed as a potential conflict of interest.

## Publisher’s Note

All claims expressed in this article are solely those of the authors and do not necessarily represent those of their affiliated organizations, or those of the publisher, the editors and the reviewers. Any product that may be evaluated in this article, or claim that may be made by its manufacturer, is not guaranteed or endorsed by the publisher.
